# Relationships between environmental variables and spatial and temporal distribution of jack mackerel (*Trachurus japonicus*) in the Beibu Gulf, South China Sea

**DOI:** 10.7717/peerj.12337

**Published:** 2021-11-04

**Authors:** Yuting Feng, Haiyi Shi, Gang Hou, Hui Zhao, Changming Dong

**Affiliations:** 1 Guangdong Ocean University, Faculty of Chemistry and Environment Science, Zhanjiang, China; 2 Southern Marine Science and Engineering Guangdong Laboratory, Zhuhai, China; 3 Oceanic Modeling and Observation Laboratory, Nanjing University of Information Science and Technology, Nanjing, China

**Keywords:** *Trachurus japonicus*, Environment factors, Remote sensing, Spatiotemporaldistribution, Generalized additive models, Beibu Gulf

## Abstract

The jack mackerel (*Trachurus japonicus*) is both a dominant pelagic fish species and an important fishing target in the Beibu Gulf, South China Sea. However, the resource status of this species fluctuates dramatically, and it has recently been added to a “red list” of threatened species of the International Union for Conservation of Nature (IUCN). Despite its economic importance and decreasing population status, limited research on its spatiotemporal distribution has been undertaken over the last decades. In order to evaluate the most crucial factors that influence the spatiotemporal variability of *T. japonicus* and to determine GAM performance and predictability, we analyze catch per unit effort (CPUE) of *T. japonicus* from Beibu Gulf over four seasons (months) from 2013 to 2014. A generalized additive model (GAMs) is populated with water depth and remotely sensed sea surface temperature (SST), sea surface salinity (SSS), sea surface chlorophyll-a concentration (Chl-a) and sea level anomaly (SLA). The CPUE of *T. japonicus* varies seasonally, with higher CPUE in summer and autumn than in spring and winter, and the highest CPUE in summer. GAM results explain 57% of the deviation explained in CPUE, with the most important variables being SLA, Month, Depth, SSS, and SST , each explaining 21.2%, 18.7%, 10.7%, 5.1%, and 1.3% of the variation in CPUE, respectively. This species occurs mainly between 50 and 75 m depth, SSS values 32.3–33.5 PSU and SST 25–30.5 °C. High CPUE sites occur near SLA ≤ 0 m, on the edge of cold eddies, and there is a certain catch near the sea surface with SLA ≥ 0 m. The spatial and temporal distribution of *T. japonicus* is affected by the season and the marine hydrological environment. This study might contribute to a better understanding of the distributional patterns of *T. japonicus* as well as provide a basis for sustainable management in the Beibu Gulf.

## Introduction

Small pelagic fishes constitute some of the most economically valuable fishery resources, account for almost half of the total global marine catch, and play extremely important roles in marine ecosystems (Peck et al., 2020; Shi & Chen, 2019). Recent perennial disturbances (*e.g.*, climate change, water extraction, overfishing, habitat degradation) have contributed to degradation of fishery resources worldwide (Bland et al., 2018; Chen et al., 2008; Pikitch et al., 2004). To ensure their sustainable use, and to develop reasonable and effective management strategies, it is necessary to understand why these pelagic fish resources vary spatially and temporally (Brehmer et al., 2007; Shi & Chen, 2019). Thus, an understanding of links between fish spatial distribution and environmental variables is essential for their conservation and fishery management (Hsieh et al., 2010; Manderson et al., 2011).

**Figure 1:**
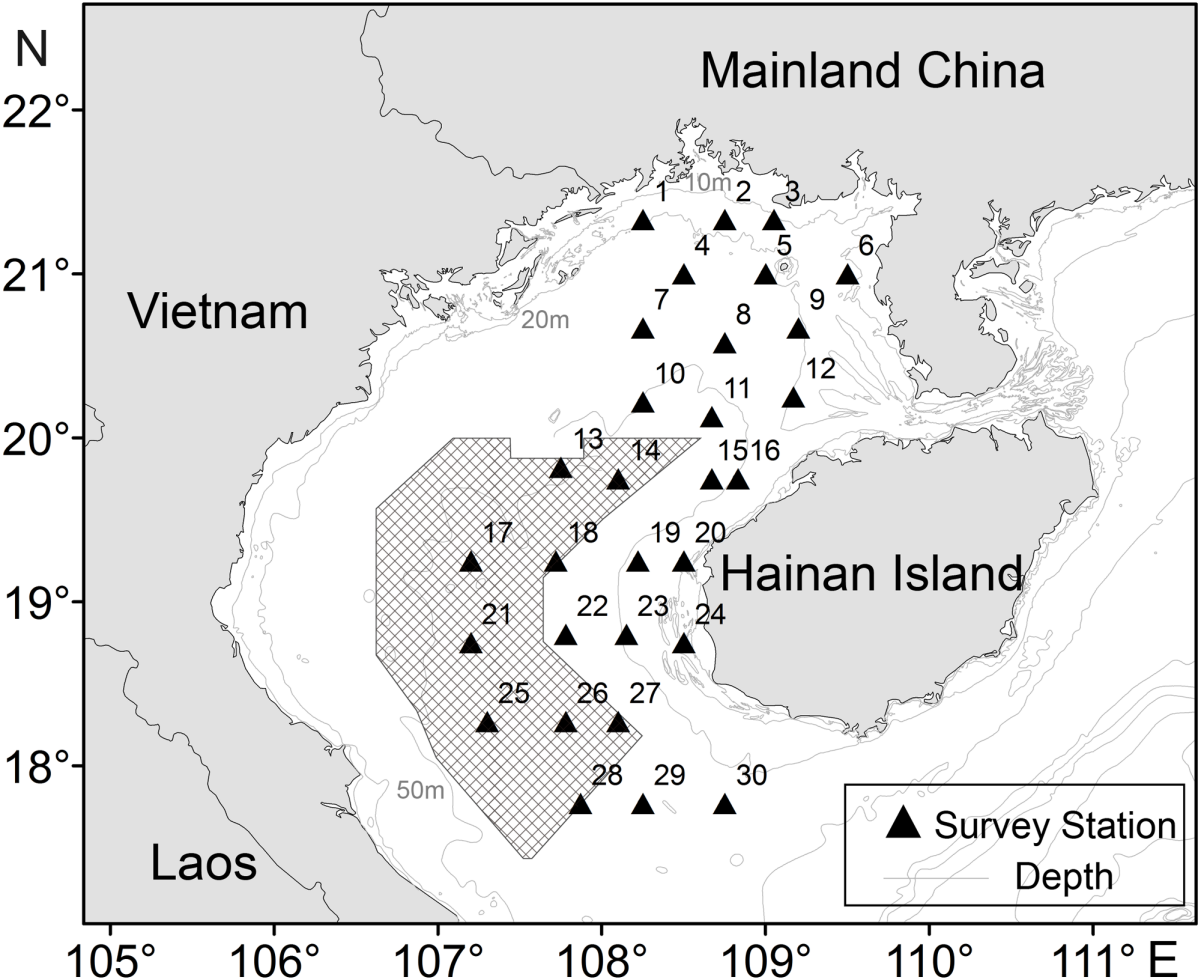
Sampling stations of *T. japonicus* in the Beibu Gulf. Shading denotes joint fishing zone (grids) between China and Vietnam. The triangle represents the study stations, and the number on the upper left represents the number of the station.

The Beibu Gulf (17–21.75°N, 105.67–110.17°E) is located in the northern South China Sea (SCS), a shared body of water between China and Vietnam, covering an area of approximately 128,000 km^2^. It is a semi-enclosed sea surrounded by territories of China, Vietnam and China’s Hainan Island (Fig. 1). With an average depth of 38 m and the maximum depth of less than 100 m. The Gulf of Beibu is known for one of China’s four major fishing grounds, and it plays a vital role in economy and employment, not only because of providing a highly productive, but also due to diverse marine fishery resources (Wang et al., 2011b). It is attributed to many estuaries (Red River, Fangcheng River, Qinjiang River, etc.) that discharge abundant nutrients from the land to the gulf (Chen et al., 2009; Qiu, Zeng & Chen, 2008). In addition, the climate around the Gulf is subtropical and monsoonal, moving northeast in April and southwest in October (Shen et al., 2020). Therefore, these basic conditions and complex marine environment provide a favorable for fish feeding, spawning, and conditioning of many species (Xia, Li & Shi, 2001).

The jack mackerel (*Trachurus japonicus*) is a warm-water pelagic fish that is widely distributed in waters of China, Korea, Taiwan, and Japan; it is an important fishery resource in the SCS, and migrates both long distances and vertically in the water column (Cao & Gao, 2006). An opportunistic consumer of (primarily) crustaceans, copepods and other micronekton, this species plays an important regulating role in regional food webs (Lin et al., 2006; Yan et al., 2012). The Beibu Gulf is one of the most productive areas in the SCS in terms of fishery resources (Cai et al., 2018), although resource density and fishery composition have declined over the last decade, with stocks of *T. japonicus* also overexploited (Li, Qiu & Wang, 2007; Qiu, Lin & Wang, 2010; Shen & Heino, 2014). It was listed as endangered (EN) in a recent IUCN red list due to threats of overexploitation (Klunzinger & Walker, 2014; Wang et al., 2020a). Because relationships between the spatiotemporal distribution of *T. japonicus* and environment are not well understood, we investigate them to provide information to enable more sustainable management and conservation of this species (Geng et al., 2018).

The choice of an analytical method is important when quantitively studying relationships between fishery resources and environment variables (Niu, Li & Xu, 2010). GAMs (generalized additive models) were useful to explore relationships between fishery resources and environmental variables among various climatic and oceanographic forcing (Febrero-Bande & González-Manteiga, 2013; Pearce et al., 2011; Zhu, 2012). GAMs is a data–driven model mostly depended on the relationship between response variables and predictors rather than structures assumed in response variables and predictors (Yee & Mitchell, 1991). Regarding model performance and stability, the predictions of GAMs were more stable with lower standard errors in R^2^ comparing with artificial neural networks (Luan et al., 2018). Thus, it may be more populated and robust for modeling and the spatial distribution of species (Leathwick, Elith & Hastie, 2006; Schmiing et al., 2013). Meanwhile, GAMs are regarded as informative tools in fisheries management, and they have been widely used in recent years (Auth et al., 2011; Choi, Min & Soh, 2021; Hua et al., 2019; Knutsen et al., 2007; Liu et al., 2019). While quantitative relationships between fishing grounds and environmental factors have used GAMs (Arcos, Cubillos & Núez, 2001; Chen & Tian, 2007; Cornic & Rooker, 2018; Feng et al., 2021; Maxwell et al., 2012; Yu et al., 2019; Hou et al., 2021), few studies have investigated relationships between *T. japonicus* fishing grounds and environmental factors in the Beibu Gulf.

We aim to (1) determine the seasonal distribution of *T. japonicus* in Beibu Gulf; (2) analyze relationships between catch per unit effort (CPUE) and environmental variables; and (3) appraise the value of this information for sustainable fishery exploitation and management.

## Materials and Methods

### Data collection

CPUE data for *T. japonicus* in Beibu Gulf (105.67–110.17°E, 17–21.75°N) was derived from fishery surveys in autumn (November) of 2013, and winter (February), spring (May), and summer (August) of 2014. Some 25 sites were sampled in each survey (Fig. 1) using a 441 kw vessel and bottom trawl of 38.5 m corkline length and 20 mm cod-end mesh. At each site, a trawl was towed for 2 h at a speed of 3 kn.

Shading denotes joint fishing zone (grids) between China and Vietnam. The triangle represents the study stations, and the number on the upper left represents the number of the station.

### Environment and geographic data

Satellite remote sensed sea surface temperature (SST) and sea surface chlorophyll-a (Chl-a) data were derived from the Moderate Resolution Imaging Spectroradiometer (MODIS). Sea surface salinity (SSS) data was downloaded from the Copernicus Marine Environment Management Service (CMES), and that for sea level anomalies (SLA) from Archiving, Validation, and Interpretation of Satellite Oceanographic (AVISO). Meridional (V) and zonal (U) current velocities were obtained from the Ocean General Circulation Model For the Earth Simulator (OFES). Depth data were derived from Google Earth elevation data (Table 1).

**Table 1:** Source of environment data. Source of datasets, temporal and spatial resolution.

**Data sources**	**Variable (unit)**	**Spatial resolution**	**Temporal resolution**
MODIS	SST (°C)	1/12° × 1/12°	8 days
MODIS	Chla (mg m^−3^)	4 km	8 days
CMEMS	SSS (PSU)	1/12° × 1/12°	monthly
AVISO	SLA (m)	0.25° × 0.25°	daily
OFES	U (m s^−1^)	0.1° × 0.1°	monthly
OFES	V (m s^−1^)	0.1° × 0.1°	monthly
Google Earth	Depth (m)	8.85 m	16 levels (elevation)

MATLAB software was used to extract SST, SSS, Chl-a, and SLA data from 2013 to 2014. Null were discarded and the data then averaged for each month. ArcGIS 10.3 (Esri, Redlands, CA, USA) was used to resample SST, SSS, and Chl-a data at different spatial resolutions and convert it into environmental data with a resolution of 0.25°. Remote sensed data are interpolated by ordinary Kriging using ArcGIS 10.3 software to plot a monthly images (Yu et al., 2018).

### Catch and effort data

(1) \begin{eqnarray*}CPUE=C/(a\cdot b)\end{eqnarray*}

where, CPUE is density of *T. japonicus* by a trawl (unit: kilograms per square kilometer), *C* is the average catch per hour in a 0.25° × 0.25° (unit: kilograms per hour), *a* is the swept area per hour, and the swept width of the trawl is taken as 2/3 of the topline length (unit: Kilometers squared per hour), *b* is the catchability coefficient with a value of 0.5.

### GAMs fitting procedures

GAMs is a nonparametric extension of the generalized linear model, which can deal directly with nonlinear relationships between response variables and multiple explanatory variables (Guisan, Edwards & Hastie, 2002). We use GAMs to analyze *T. japonicus* CPUE data and environmental factors. The

general expression of GAM is:

(2) \begin{eqnarray*}\mathrm{Y }=\alpha +\sum _{\mathrm{j}=1}^{\mathrm{n}}{\mathrm{f}}_{\mathrm{ i}} \left( {\mathrm{x}}_{\mathrm{j}} \right) +\end{eqnarray*}

where Y = the CPUE (kg/km^2^); x_j_ = x_j_ the explanatory variable (the spatiotemporal and environmental factors for each site); *α* = the intercept that fits for the function; *ɛ* = the residual error; and f_i_(x_j_) = a one-variable function of the independent variable (a spline smoothing function). We use the mgcv package in R 4.0.5 software to construct and test the model (Pedersen et al., 2018; Wood, 2006). To determine the expression form of the GAM, A stepwise method was used to select variables that have a significant influence on the model (Wood, 2004; Wood, 2011).

### Model test

(1) The Akaike information criterion (AIC) was used to test the fit of the model after the gradual addition of factors; the smaller the value, the better the fit of the model (Burnhan & Anderson, 2002). Generalized cross validation (GCV) was used to evaluate predictive variables of the model; the smaller the value, the better the modeling ability (Shchetinnikov, 1992; Stone, 1985). Chi-square and F-tests were used to evaluate the nonlinear contribution of nonparametric effects and assess the significance of each factor (Stone, 1985). AIC is calculated as follows (Venables & Dichmont, 2004):

(3) \begin{eqnarray*}\mathrm{AIC}\hspace*{2.22198pt}=\theta +\hspace*{2.22198pt}2\mathrm{df}\varphi \end{eqnarray*}

where *θ* = the deviation; *df* = the effective degree of freedom; and *φ* = the variance. (2) The W’ normal test method were proposed by Shapiro et al. (Shapiro & Francia, 1972). The normal quantile plot was used to examine the distribution of GAM residuals, to indicate the distribution of model residuals and the histogram of residuals. A VIF (variable inflation factor) test suggested that factors without collinearity have VIF values <5 (Cornic & Rooker, 2018), except for Lat (VIF > 5) (Table 2).

**Table 2:** VIF analysis. VIF test between environmental factors.

**Factors**	**VIF**
Lon	3.998
SLA	2.670
SSS	2.852
SST	1.590
Chla	3.618
Depth	3.500
Distance	4.003

### Center of gravity

The center of gravity (CoG) method was used to analyze spatial and temporal variability in CPUE (Chen et al., 2003). The CoG of *T. japonicus* CPUE is calculated as follows:

(4) \begin{eqnarray*}\mathrm{X}=\sum _{\mathrm{i}=1}^{\mathrm{K}} \left( {\mathrm{C}}_{\mathrm{ i}}\times {\mathrm{X}}_{\mathrm{i}} \right) /\sum _{\mathrm{i}=1}^{\mathrm{K}}{\mathrm{C}}_{\mathrm{ i}}\end{eqnarray*}

(5) \begin{eqnarray*}\mathrm{Y }=\sum _{\mathrm{i}=1}^{\mathrm{K}} \left( {\mathrm{C}}_{\mathrm{ i}}\times {\mathrm{Y }}_{\mathrm{i}} \right) /\sum _{\mathrm{i}=1}^{\mathrm{K}}{\mathrm{C}}_{\mathrm{ i}}\end{eqnarray*}

where X and Y = the longitude and latitude of the CoG; *C*_*i*_ C_i_ = the yield of fishing area *i*; X_i_ and Y _i_ = the central longitude and latitude of fishing area *i*; and K = the total number of fishing areas.

## Results

### Seasonal variation in *T. japonicus* CPUE

**Figure 2:**
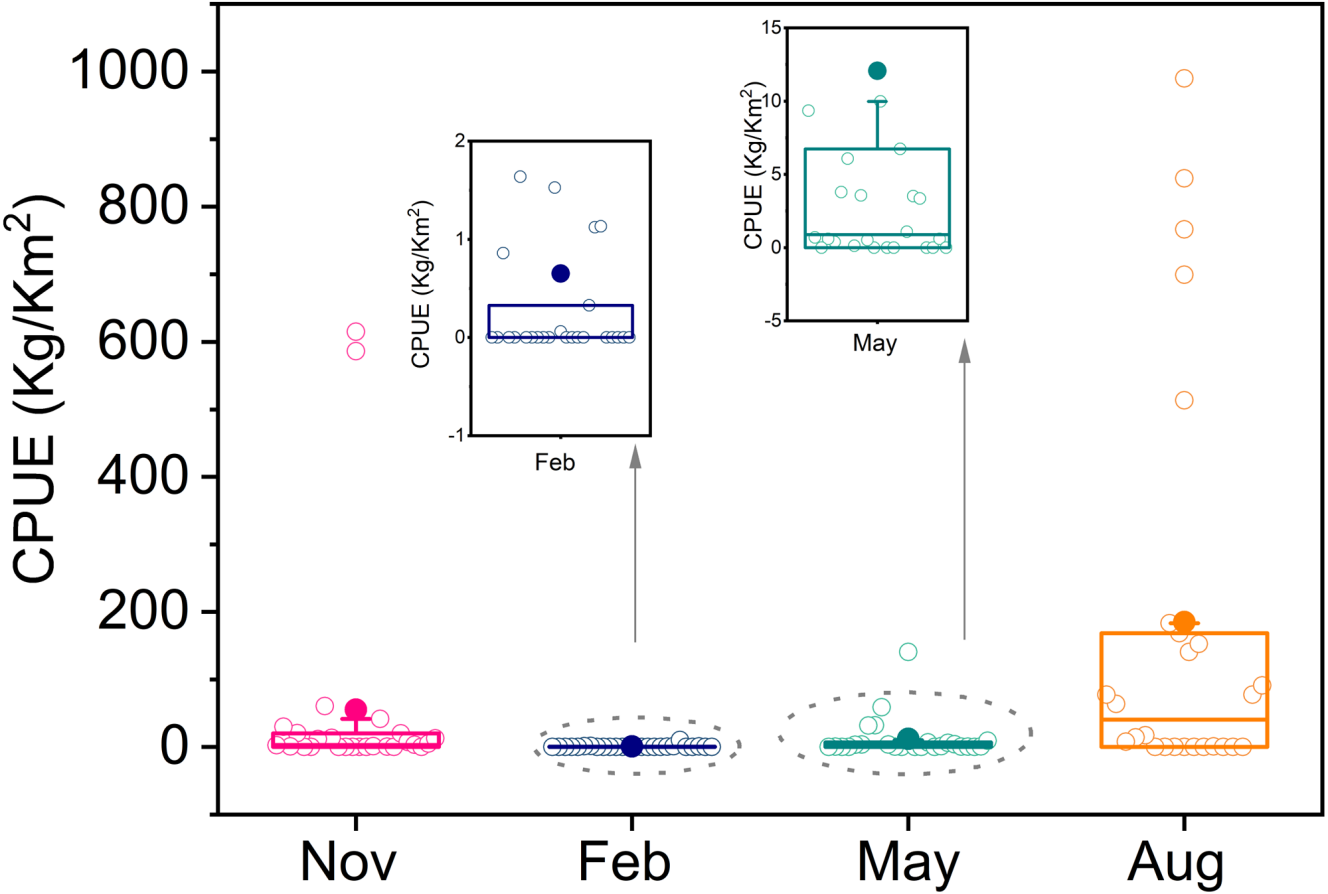
Boxplot of resource density in four seasons.

*T. japonicus* is widely distributed, and its density varied seasonally from autumn 2013 to winter 2014. Average resource density was highest in summer (184.8 kg/km^2^), then autumn (55.1 kg/km^2^), and lowest in winter (0.7 kg/km^2^). There was an abnormally high values in summer, of which the highest was 985.6 kg/km^2^ (Fig. 2).

### GAMs analysis

**Figure 3:**
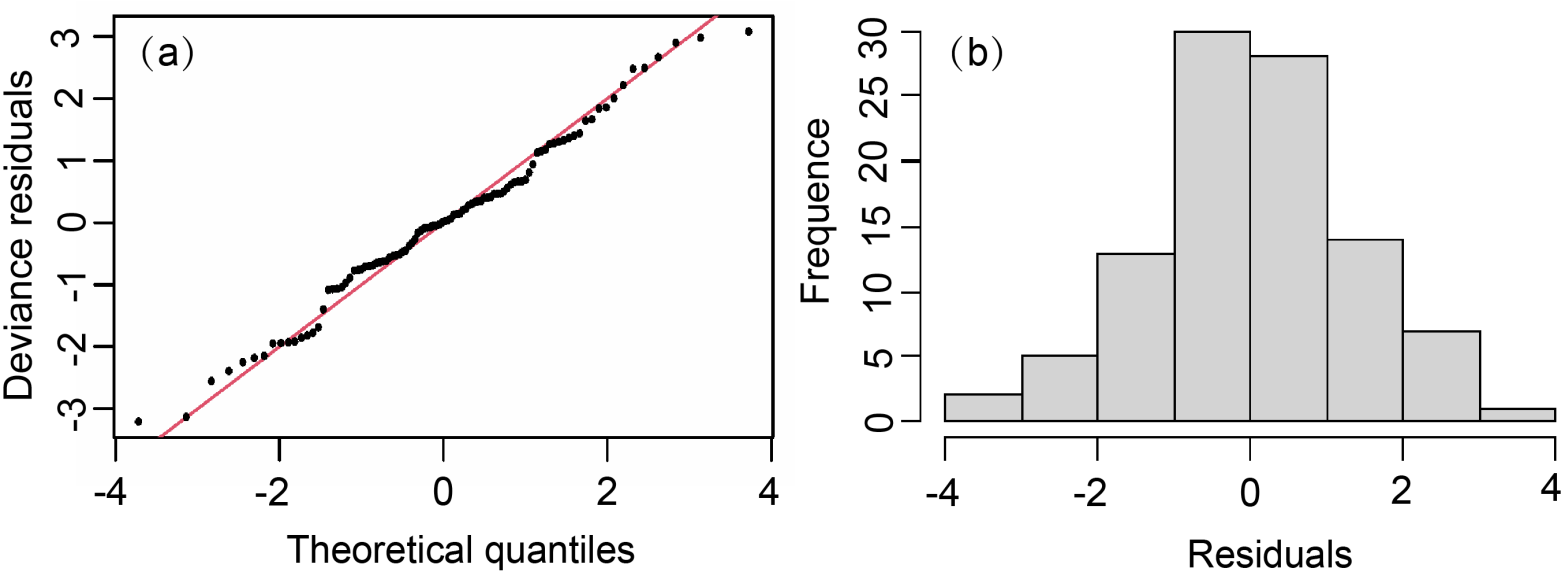
Normal quantile plot and histogram of residuals of GAM residual. (A) Normal quantile plot. (B) Histogram residuals of GAM.

The model showed a skew normal distribution (Fig. 3), with a W′ statistic of 0.987 (<0.05), which reached the 95% reliability level of statistical significance. The residuals passed the normality test and adequately conformed to a normal distribution. Except for Lat (VIF > 5), we added all factors to the model. The importance of each variable was selected based on AIC and GCV values. Our GAM formula was

(6) \begin{eqnarray*}\log \nolimits (Y+1)=s(Month)+s(SLA)+s(Depth)+s(SST)+s(SSS)+\end{eqnarray*}

where *Y* = *T*. *japonicus* density (because of 0 CPUE values, we log transformed our data after adding 1 (*Y* + 1) (Guan et al., 2009; Porch, 1995); *s* = the natural spline smoothing function; *s*(*Month*) = the effect of month; s(*SLA)* = the effect of sea level anomaly; *s*(*Depth*) = the effect of depth; *s*(*SST*) = sea surface temperature; *s*(*SSS*) = the effect of sea surface salinity; and *ɛ* = modeling error, which conforms to a Gaussian distribution. The cumulative explanatory bias of GAM on *T. japonicus* was 57%, with an R^2^ of 0.51 (Table 3).

**Table 3:** Test of GAM for modeling CUPE *T. japonicus* and the corresponding model factors.

**Model factors**	**Residual deviance**	**Adjusted** **R** ^**2**^	**AIC**	**GCV**	**Deviance explained (%)**
Log (Y + 1) = NULL	417.52	0	430.70	4.26	0
Log (Y + 1) = s(Month)	339.28	0.17	413.71	3.60	18.7
Log (Y+1) = s(Month) + s(SLA)	251.07	0.37	387.57	2.78	39.9
Log (Y+ 1) = s(Month) + s(SLA) + s(Depth)	206.41	0.46	376.43	2.50	50.6
Log (Y + 1) = s(Month) + s(SLA) + s(Depth) + s(SST)	200.66	0.47	374.57	2.45	51.9
Log(Y+1) = s(Month)+ s(SLA) + s(Depth) + s(SST) + s(SSS)	179.39	0.51	372.48	2.43	57

Results of GAM fitting indicate the best explained CPUE of *T. japonicus* included five explanatory variables (Tables 3, 4), of which SLA was the most important, explaining contributing 21.2%, followed by Month, Depth, SSS, and SST, contributing 18.7%, 10.7%, 5.1%, and 1.3%, respectively. Excepting SSS, an ANOVA F-test indicated that all model factors were significant (*P* < 0.05). However, when SSS was added to the model, AIC values decreased further, and the cumulative deviation interpretation increased, which indicates increased model fit. Therefore, SSS was kept in the model. A Chi-square test revealed the nonparametric smoothing effect of predictive variables, According to Chi-square tests, Month, SLA, Depth, and SST had the best nonparametric smoothing effects, while that of SSS was lower (Table 4).

**Table 4:** Contributions of the selected variables in GAMs.

**Variables**	**d.f.**	**Contribution (%)**	**Pr (F)**	**Pr (Chisq)**
SLA	2.67	21.2	0.00^***^	0.00^***^
Month	1.85	18.7	0.00^***^	0.00^***^
Depth	2.94	10.7	0.00^**^	0.00^**^
SST	1.00	1.3	0.03^*^	0.03^*^
SSS	4.67	5.1	0.1	0.1

**Notes.**

*** *p* < 0.001.

** *p* < 0.01.

* *p* < 0.05.

Pr (F) *p*-value from an ANOVA F-ratio test Pr(chisq) a type of score test to evaluate the nonlinear contribution of non-parametric effects SLA sea level anomaly SST sea surface temperature Lat Latitude Lon Longitude Distance offshore distance

**Figure 4:**
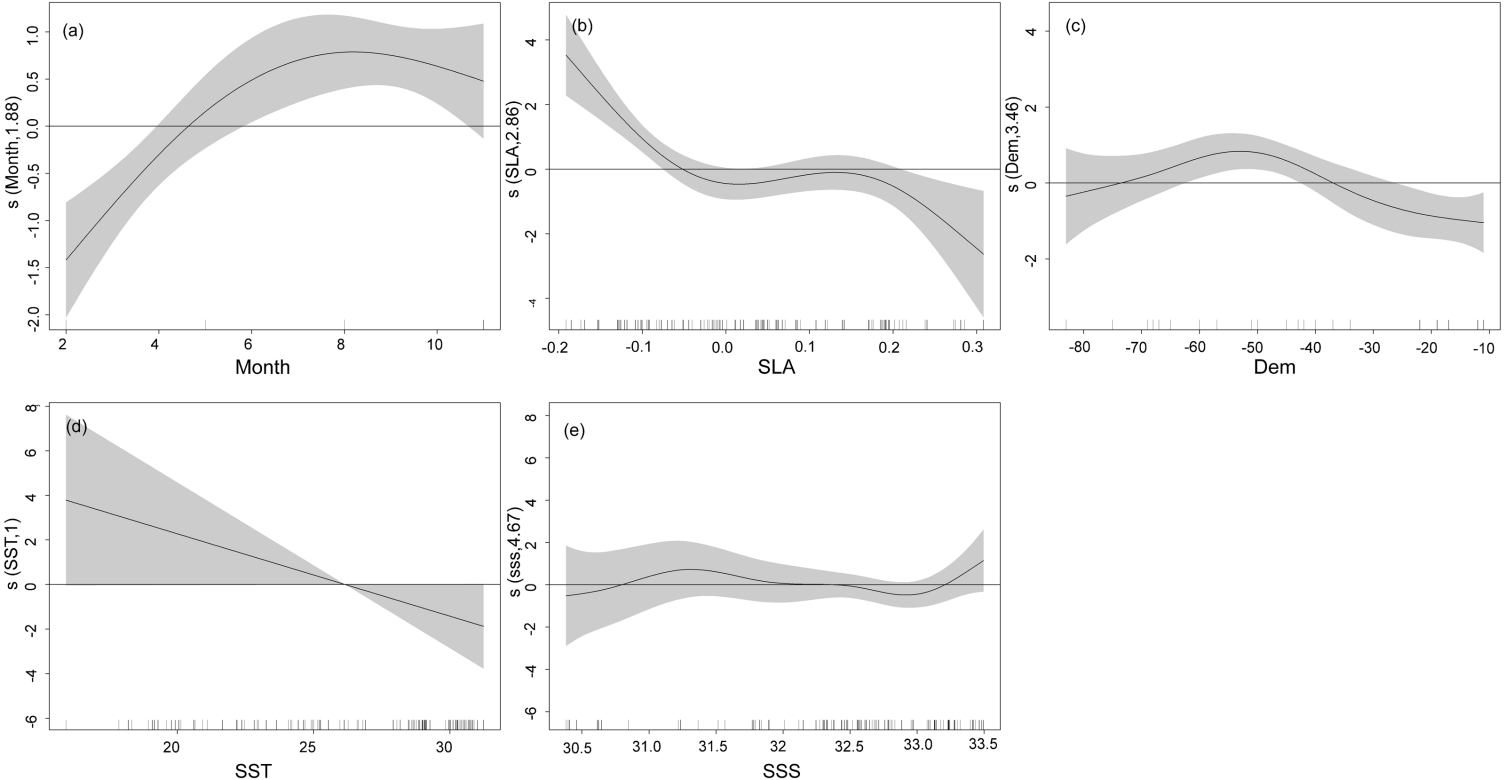
GAM analysis on the impact of each factor on *T. japonicus* CPUE: (A) Month, (B) sea level anomaly, (C) Depth, (D) sea surface temperature, (E) sea surface salinity. Gray-shaded areas representing the 95% confidence interval with the rug plot on the *x*-axis representing the actual fishing data points, and the solid curve shows the fitted GAM functions describing the effect of environmental variables on the response variables (CPUE). GAM indicates a positive effect of environmental variables on CPUE when the fitted solid curve of the GAM function was above the zero axis.

The nonlinear relationship between environmental variables and CPUE is shown in Fig. 4. Generally, the narrower the confidence interval the higher a correlation. Relationships between CPUE and explanatory variables from GAMs revealed the highest CPUE of *T. japonicus* occurred in summer, with a significant increase from winter to summer. A positive effect of season on CPUE was also observed between spring and summer, but effects were negative from winter to spring (Fig. 4A). A positive effect of SLA on CPUE occurred below −0.05 m, but negative effects were noted above 0.05 m (Fig. 4B). A positive effect on CPUE was obvious between 35 and 75 m depth, while negative effects occurred below 40 m and above 75 m (Fig. 4C). The effect of SST on *T. japonicus* CPUE maintained a negative linear correlation between 16 and 32 °C; Below 28 °C was a positive effect, while above 28 °C was a negative effect (Fig. 4D). Salinity from 30.0 to 32.5 PSU had a positive effect on CPUE (Fig. 4E).

### Relationship between oceanographic factors and *T. japonicus* CPUE

**Figure 5:**
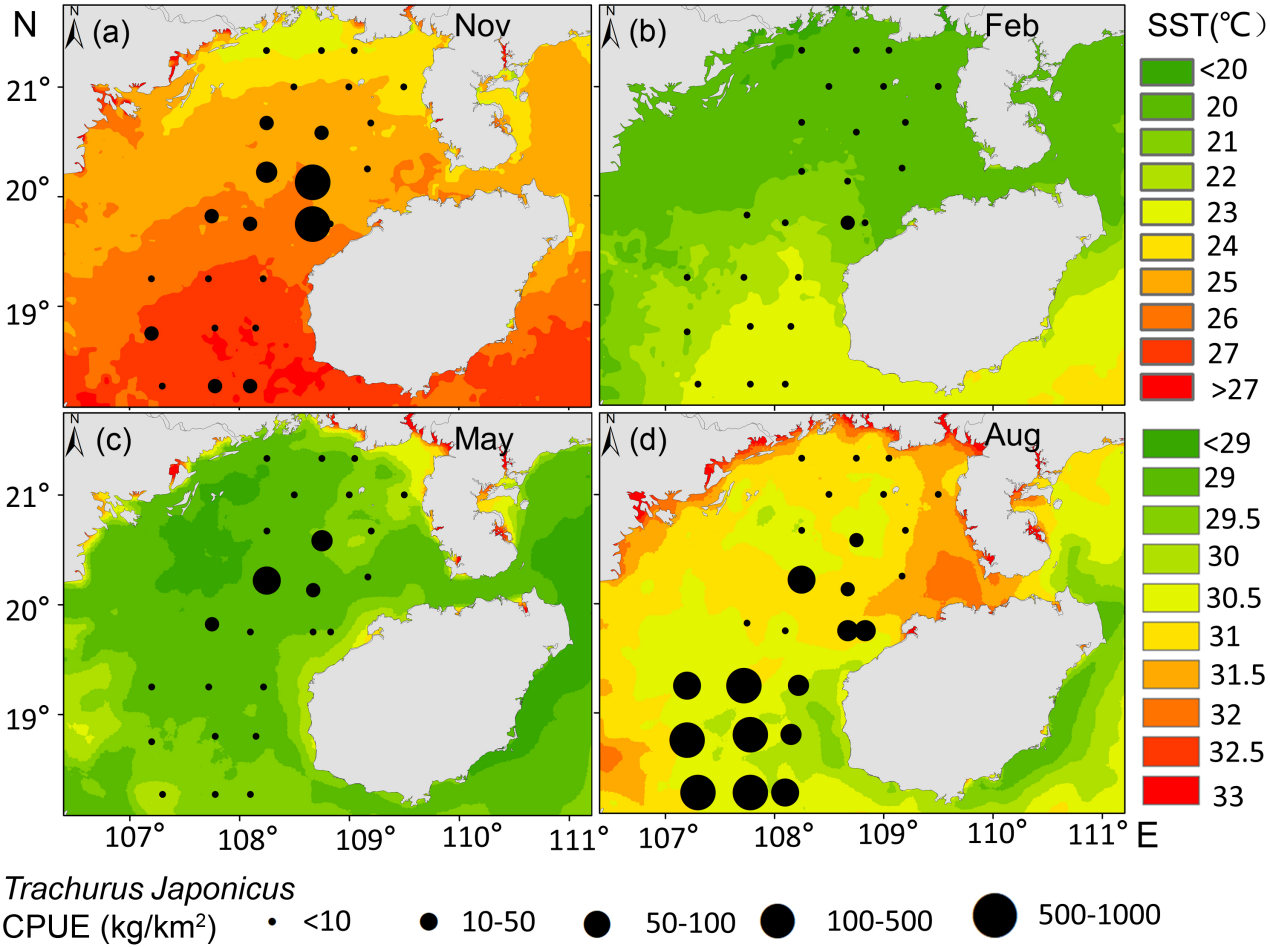
Relationship between SST and CPUE. (A) Autumn SST, (B) Winter SST, (C) Spring SST, (D) Summer SST, and *T. japonicus* CPUE.

**Figure 6:**
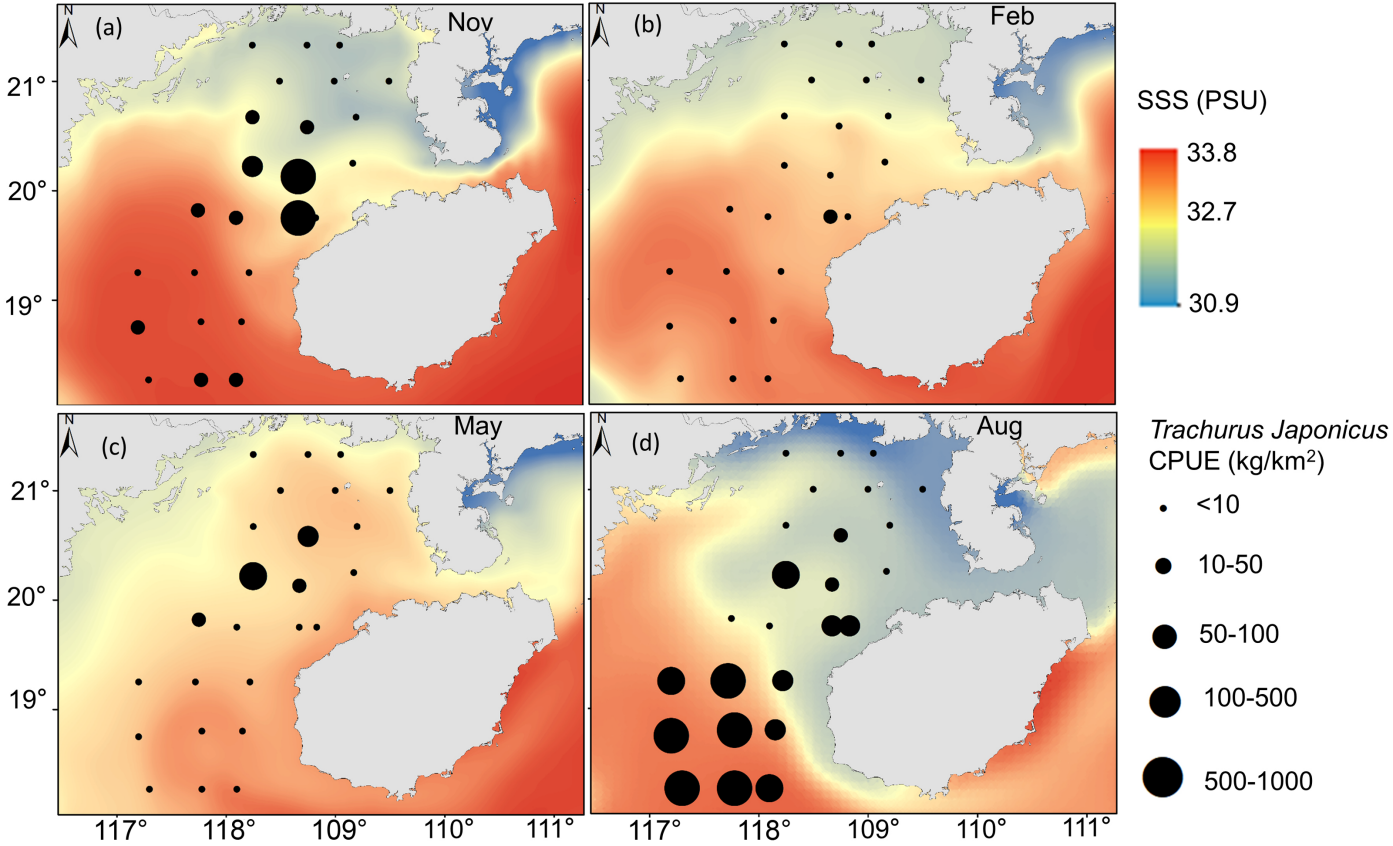
Relationship between SSS and CPUE. (A) Autumn SSS, (B) winter SSS, (C) Spring SSS, (D) Summer SSS, and *T. japonicus* CPUE.

*T. japonicus* fishing grounds in the Beibu Gulf are concentrated mainly in waters of 25–30.5 °C SST and SSS of 31.2–33.5 PSU. Suitable SST varies from spring (28.1–29.1 °C, Fig. 5C), to summer (30–30.7 °C, Fig. 5D), to autumn (24.5–25.2 °C, Fig. 5A), and winter (20.6–21 °C, Fig. 5B). Most suitable SSS values for *T. japonicus* in spring are 32.5–32.7 PSU (Fig. 6C), summer (33.0–33.5 PSU, Fig. 6D), autumn (31.2–33.2 PSU, Fig. 6A), and winter (32.8–33.0 PSU, Fig. 6B).

**Figure 7:**
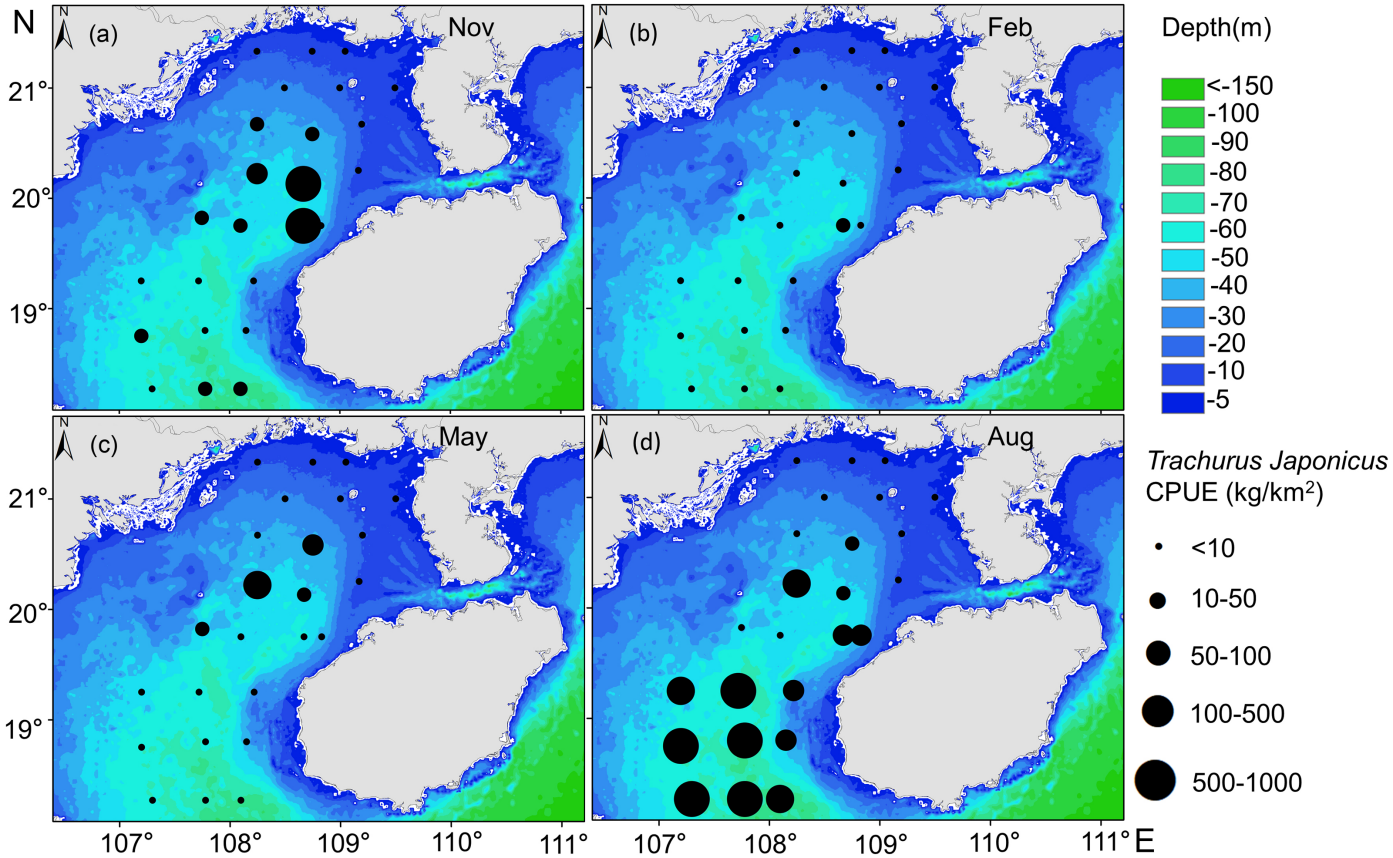
Relationship between water depth and CPUE. (A) Autumn water depth, (B) Winter water depth, (C) Spring water depth, (D) Summer water depth, and *T. japonicus* CPUE.

Relationships between the spatial and temporal distribution of *T. japonicus* CPUE and depth revealed most *T. japonicus* to occur at <85 m, and for CPUE to first increase and then decrease with increased depth (10–75 m, Fig. 7), with a maximum at 67 m (989.6 kg/km^2^). From spring (May) to winter (Feb), suitable water depth ranges for *T. japonicus* first increased and then decreased: depths between 42 m and 50 m were suitable in spring (Fig. 7C), between 65 m and 75 m in summer (Fig. 7D), 40 m and 60 m in autumn (Fig. 7A), and 51 m and 60 m in winter (Fig. 7B).

### Spatial and temporal variability in center of gravity of *T. japonicus* CPUE in the Beibu Gulf

**Figure 8:**
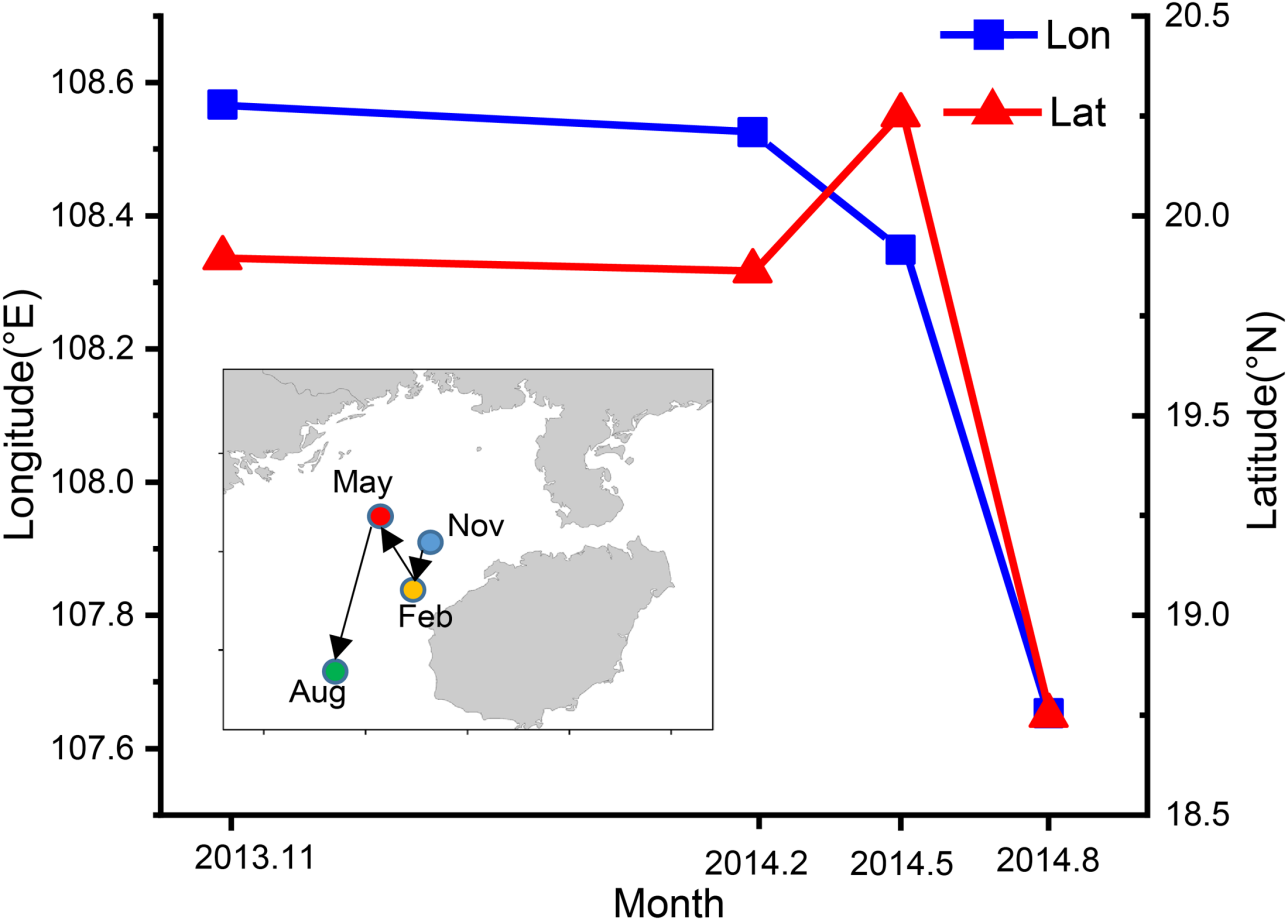
Seasonal variation of fishing ground gravity of *T. japonicus* in the Beibu Gulf. The blue line indicates the longitude and the red line represents the latitude.

The CoG shifted seasonally from the northeast to southwest (Fig. 8). In autumn the CoG was located off Lingao (19.89°N, 108.57°E), but shifted to off Dongfang (19.86°N, 108.53°E) in winter, before moving northwest in spring to off BechLongVi island (20.25°N, 108.35°E), and then to the sea area in the mouth of Beibu Gulf in (18.75°N, 107.65°E) August (Fig. 8).

The blue line indicates the longitude and the red line represents the latitude.

### The predicted spatial distribution of *T. japonicus* CPUE

**Figure 9:**
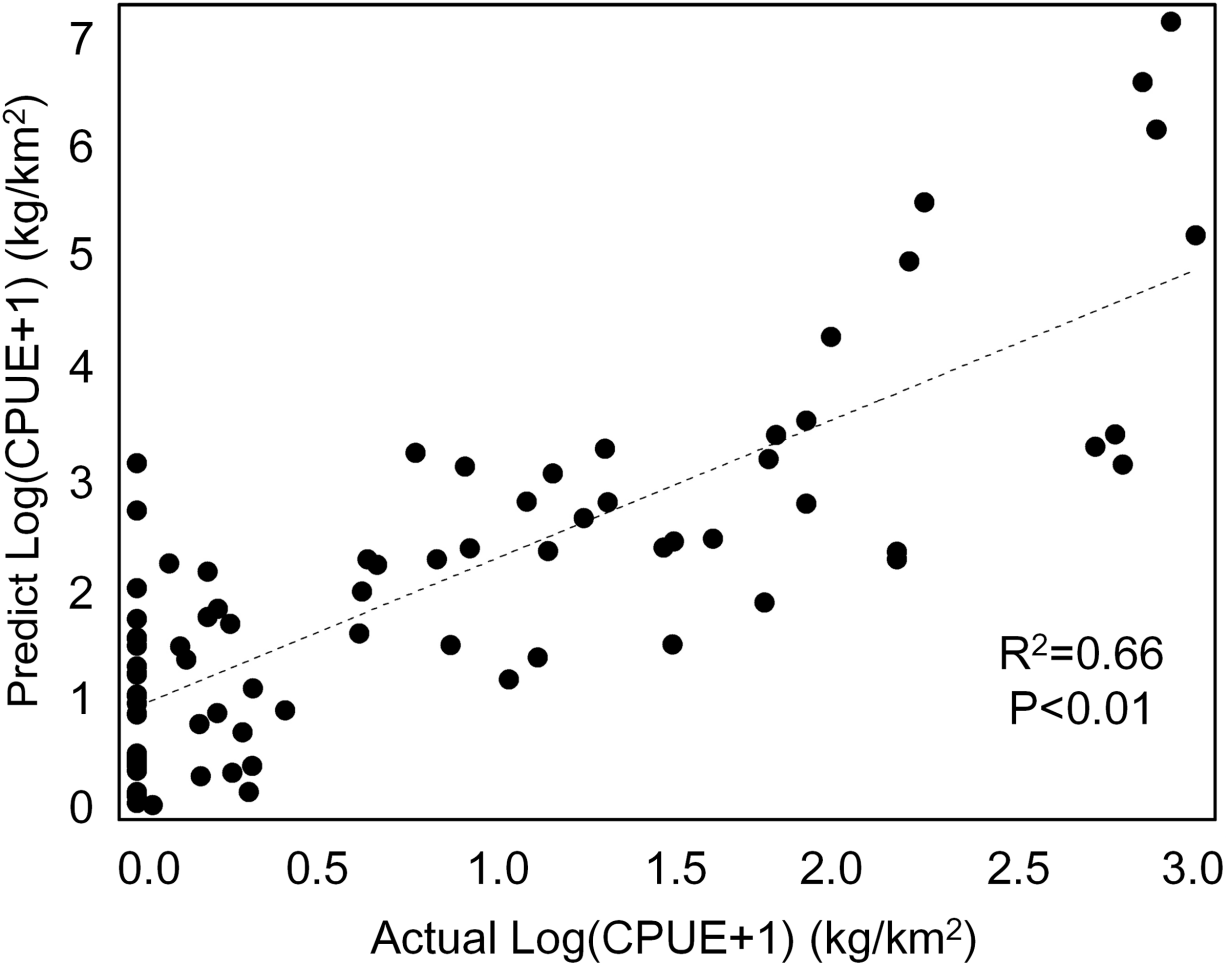
The total scatter plots of actual *versus* predicted CPUE within 2013–2014.

**Figure 10:**
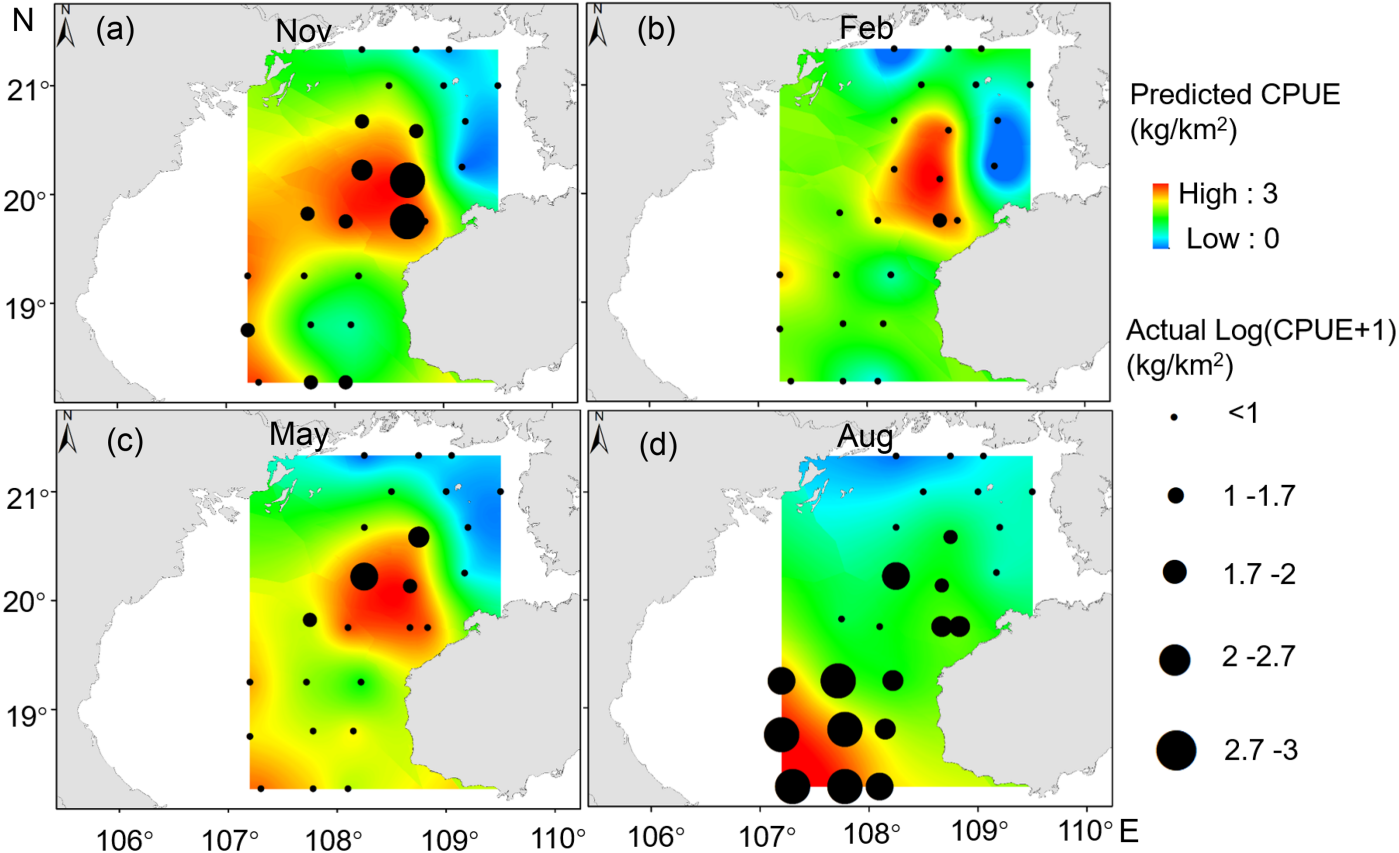
Predicted *T. japonicus* CPUE overlaid with actual fishing CPUE (black dots) from 2013 to 2014. (A) Autumn. (B) Winter. (C) Spring. (D) Summer.

Our GAM model predictions fitted well with actual CPUE (*R*^2^ = 0.66, *p* < 0.01; Fig. 9). The maximum R^2^ occurred in summer (*R*^2^ = 0.77, *p* < 0.01) and minimum R^2^ in winter (*R*^2^ = 0.05, *p* > 0.05; Fig. S1). The distribution of predicted seasonal *T. japonicus* CPUE correspond well with actual fishing CPUE (black dots in Fig. 10), and the size of symbol increases with the actual catch. Actual fishing locations largely coincide with predicted areas of high CPUE. *T. japonicus* mainly occurred in central sea areas of the Beibu Gulf from November (autumn) to May (spring), and in the mouth of Beibu Gulf in August (summer) (Fig. 10).

## Discussion

### Spatial and temporal distribution of *T. japonicus* in the Beibu Gulf

Beibu Gulf is an important spawning and feeding ground and key habit for many fish species. The location of *T. japonicus* fishing grounds changes with month (Qiu, Lin & Wang, 2010; Venables & Dichmont, 2004). We report seasonal differences in fishing grounds in the Beibu Gulf throughout the year, with the highest resource density occurring in summer, and the lowest in winter–a result that consistent with previous research (Yan et al., 2019). Month greatly influences CPUE in the Beibu Gulf (explaining18.7%; Table 4). Seasonal variation in *T. japonicus* CPUE is mainly attributed to variation in biomass of this species in the Beibu Gulf, and differences in growth rate at different temperatures (Fan, Feng & Chen, 2018). Growth and migration of fish contribute greatly to seasonal variation in fish resources in the northwest Beibu Gulf (Fu et al., 2019).

The CPUE of *T. japonicus* occurs mainly between 107.2–108.7°E and 18.3–20.2°N (Figs. 5, 6, 7). In spring, CPUE of *T. japonicus* is mainly distributed in 107.8–108.8°E and 20–21°N, possibly because the seabed is flat and the terrain inclines from °N orthwest to southeast (Figs. 5C, 6C, 7C). Many rivers along the coast transport nutrients into the sea (Song, Huang & Shi, 2004). Shallow depths and enhanced tidal currents in this area also enhance water column mixing, increasing surface nutrients, and promoting the phytoplankton growth conducive to feeding and aggregation of marine species (Wang et al., 2020b). The summer distribution of CPUE (mainly from 107.2–107.8°E) may be attributable to counter clockwise currents and enhanced northwest–southeast oriented ocean processes in the western part of the bay, influenced by southwest monsoon (Wang et al., 2011a) (Figs. 5D, 6D, 7D). SST and SSS in the bay also increased at this time, creating conditions suitable for growth of the young fish (Chen & Qiu, 2005; Yan et al., 2018). Alternatively, due to the summer rainfall increases has led to a significant expand in river runoff, bringing abundant land-sourced nutrients and bait for fish. Under the southwest monsoon, currents may have transported nutrients and plankton to the continental shelf and slope, and *T. japonicus* migrated to the southwest for feeding (Shi et al., 2019; Yan, 2019). In addition, juvenile *T. japonicus* mainly feed on planktonic crustaceans and copepods, while adult fish mainly feed on copepods, long tails and short tails (Qiu, 1980). In summer, the species of copepods generally increase from the northern community to the bay mouth. Therefore, the changes in food habits in spring and summer may also be the reason for the high CPUE in summer (Zeng et al., 2016). In autumn, *T. japonicus* occurred mainly between 108.3–108.7°E and 19.8–20.1°N and in the northern bay, possibly because temperatures in the Beibu Gulf fell, and fish within the bay migrated northeast to shallow seas to reproduce (Chen & Qiu, 2005) (Figs. 5A, 6A, 7A). In winter, a strong, stable, cold high pressure system increased the sea’s stability in northern monsoon conditions, and most fish migrated northeast or into the open sea to overwinter (Yan, 2019; Figs. 5B, 6B, 7B). The distribution and changes in *T. japonicus* CPUE appear to be significantly influenced by water masses and cyclones, and closely related to their lifespan.

### Relationship between *T. japonicus* CPUE and environmental factors

Environmental variation may affect the spatial and temporal distribution of *T. japonicus* (Sassa et al., 2014; Sassa et al., 2008). We report SST, SSS, water depth, and SLA to significantly affect the distribution of *T. japonicus* in the Beibu Gulf. Temperature and salinity affect reproduction, survival, growth, and the distribution of marine species (Sassa & Konishi, 2010; Wang et al., 2011c).

Temperature is one of the major environmental factors affecting distribution of *T. japonicus* (Cui et al., 2012; Niu et al., 2010; Niu, Li & Xu, 2010). However, SST did not contribute significantly in this study (explaining 1.3%, Table 4), which may be due to the fact that *T. japonicus* are more sensitive to changes of water temperature gradients in the northern South China Sea (Fan, Feng & Chen, 2018). In addition, it may be related to the amount of data or covered by other factors in this study. We report suitable SST conditions for this species to differ seasonally, consistent with previous research (Li et al., 2016). The SST of *T. japonicus* fishing grounds in the Beibu Gulf ranges 17–31 °C, with high CPUE occurring in waters of SST 25.0–30.5 °C mainly from 18.27 to 20.13°N (Fig. 5). Suitable water temperature for *T. japonicus* in the Beibu Gulf exceeds that in the East China Sea (21–23 °C) (Sassa & Konishi, 2010) and southeast Pacific (15 °C isotherm) (Li et al., 2016), possibly because of lower water temperatures in the latter regions.

SSS also affects *T. japonicus* CPUE (explaining 5.1%, Table 4). Salinity fronts are generally associated with monsoon winds, ocean currents, topography, and boundaries between water masses (Chen, 2009), and across them temperature, salinity and other oceanographic parameters can change rapidly (Chen, Wang & Guo, 2006). These fronts are characterized by mixing, string, enhanced productivity, and ecotones (Park, Chung & Kim, 2004; Park & Chu, 2006). We report fishing locations to concentrate near salinity fronts, particularly in summer and autumn (Figs. 6D, 6A), possibly because microalgae and zooplankton accumulate in these areas, and food is abundant (Bertrand et al., 2002). Water temperature and salinity may also shift with climate change (Bell et al., 2013), which ultimately will affect the spatial and temporal distributions of *T. japonicus*.

Depth affects *T. japonicus* CPUE (explaining 10.7%, Table 4). Depth directly affects temporal and spatial changes in hydrological elements, especially temperature, salinity, and current velocity. Moreover, as a generalization, increased depth is associated with decreased variation in each of these parameters (Chen, 2004). We report the highest CPUE of *T. japonicus* to occur mainly between 50 and 75 m near the mouth of Beibu Gulf, possibly because deeper waters provide a more favorable habitat for this species (Yan et al., 2019).

When SLA is added into the GAM model, the model AIC value decreases and its explaining increases (explaining 21.2%, Table 4), which indicates that this variable is an important one to monitor with respect to *T. japonicus* fishing ground locations. SLA is the characterization of seawater dynamics (Xu et al., 2019a). Generally, surface flow fields corresponding to positive or negative SLA values represent anticyclonic or cyclonic distributions, respectively (Ansorge et al., 2015; Dong et al., 2014). We report high *T. japonicus* CPUE along the edges of anticyclonic and cyclonic eddies, and low CPUE within anticyclonic eddies (Figs. S2A, S2D). This may be due to higher chlorophyll concentrations at the edge of anticyclonic eddies than inside the eddies (Gaube et al., 2015). In addition, surface waters within anticyclonic eddies are irradiated and subject to downward flow caused by eddy pumping, which is nutrient-poor and has low chlorophyll concentration (Yang et al., 2020). The *T. japonicus* CPUE peaked at SLA around −0.2 m and 0.2 m, with a maximum value at −0.2 m (Fig. 4B), possibly because high fishing grounds often occur in areas of sea between high and low SLA values (Guan et al., 2009), which may be related to fronts formed by cold and warm eddies (Yang et al., 2019). It is also possible that in waters near the intersection of sea surface height anomalies >0 m and <0 m that the mixing and stirring of warm and cold water masses cause nutrients and chlorophyll from deeper waters to reach the surface, which provides abundant food for *T. japonicus* (Guo et al., 2020). Negative sea surface height values also indicate the convergence or shearing of currents, enriched nutrients and increased Chl-a (Xu et al., 2019b), and an abundance of food for fish. We speculate that currents (*e.g.*, eddies, upwelling) at SLA values from −0.2 m to 0.2 m provide favorable conditions for *T. japonicus* but negatively affect *T. japonicus* CPUE at SLA >0.2 m. We report no positive effect of Chl-a concentration on CPUE of *T. japonicus*, which may be related to the feeding habits (Niu et al., 2010), growth (Niu, Li & Xu, 2010), and developmental stages of *T. japonicus* in the Beibu Gulf, which is consistent with previous research (Arcos, Cubillos & Núez, 2001).

### Fishery management and future improvements

Ecosystem-based fishery management (EFM) is considered essential for sustainable fishery management (Dolan, Patrick & Link, 2015; Pikitch et al., 2004). Implementation of this approach requires knowledge of the impacts of fisheries on the environment (Kellner et al., 2011; Link & Browman, 2014). The Beibu Gulf as a key habitat for nearly 500 fish species (Wang et al., 2011b). we should establish a systematic marine management and suitable habitat mechanism of fishing grounds as soon as possible. Seeking interdisciplinary cooperation from causes as well as impacts of Catch rates trend downward to provide a powerful support for the conservation of fishery resources in the Beibu Gulf.

Our GAM is based on data for four months only considers the general environmental factors. But fishing grounds for *T. japonicus* are closely related to biological, physical and chemical factors (Xu & Zhang, 2007), further biotic and abiotic predictor variables, such as dissolved oxygen and El Niño & La Niña events, should be taken into consideration in future studies.

## Conclusion

In this research, satellite remote sensing data of SST, SSS, SLA, Chl-a, water depth and fishery resource production were used to analyze the relationship between *T. japonicus* and marine environments in the Beibu Gulf in 2013–2014.

1. Positive effects on *T. japonicus* CPUE were observed for SLA of below −0.05 m and water depth of 35–75 m, SST of below 28 °C, SSS of 30.8 to 31.8 PSU, and negative effects were noted for SLA of above −0.05 m and water depth of below 40 m and above 75 m, SST exceeds 28 °C.

2. The GAM indicated the importance of SLA and Depth of the hydrological environment to the variability of spatial and temporal variability of *T. japonicus* in the Beibu Gulf.

3. The GAM model fitted well between the predicted and actual CPUE, showing the reliability and accuracy to indicate the distributional pattern for *T. japonicus* in the Beibu Gulf.

##  Supplemental Information

10.7717/peerj.12337/supp-1 Supplemental Information 1 Raw data

10.7717/peerj.12337/supp-2 Supplemental Information 2 The scatter plots of actual *versus* predicted CPUE within 2013-2014 (A) Autumn. (B) Winter. (C) Spring. (D) Summer

10.7717/peerj.12337/supp-3 Supplemental Information 3 Relationship between current and CPUE (A) Autumn. (B) Winter. (C) Spring. (D) Summer

## Additional Information and Declarations

### Data Availability

The following information was supplied regarding data availability: The raw measurements are available in the Supplementary File.
