# Supplementary figures and images for: Relationships between environmental variables and spatial and temporal distribution of jack mackerel (Trachurus japonicus) in the Beibu Gulf, South China Sea
Source: PeerJ. 2021 Nov 4;9:e12337. doi: 10.7717/peerj.12337 (PMC8572524; doi:10.7717/peerj.12337)

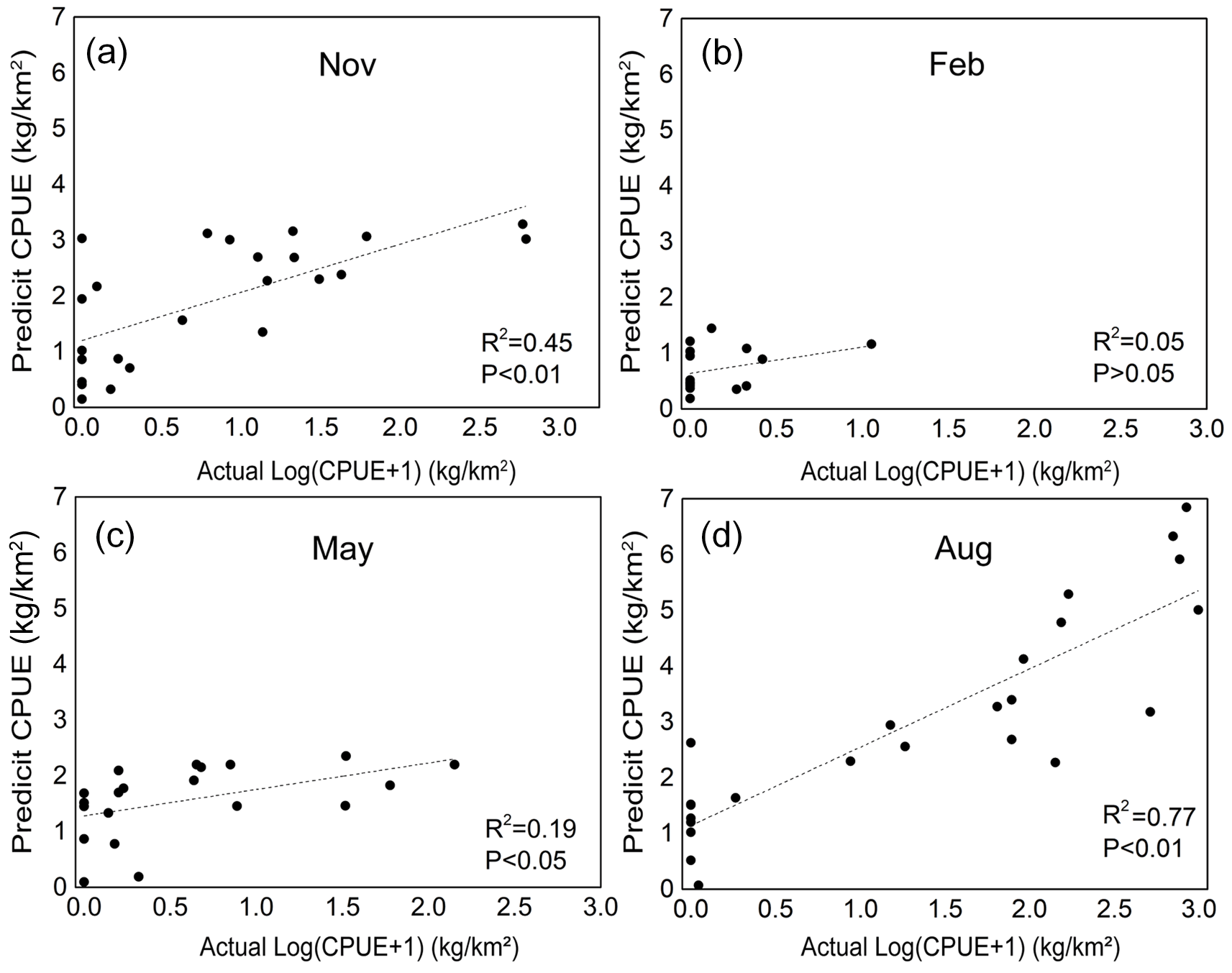

Supplement: Supplemental Information 2 — (A) Autumn. (B) Winter. (C) Spring. (D) Summer [file peerj-09-12337-s002.png]

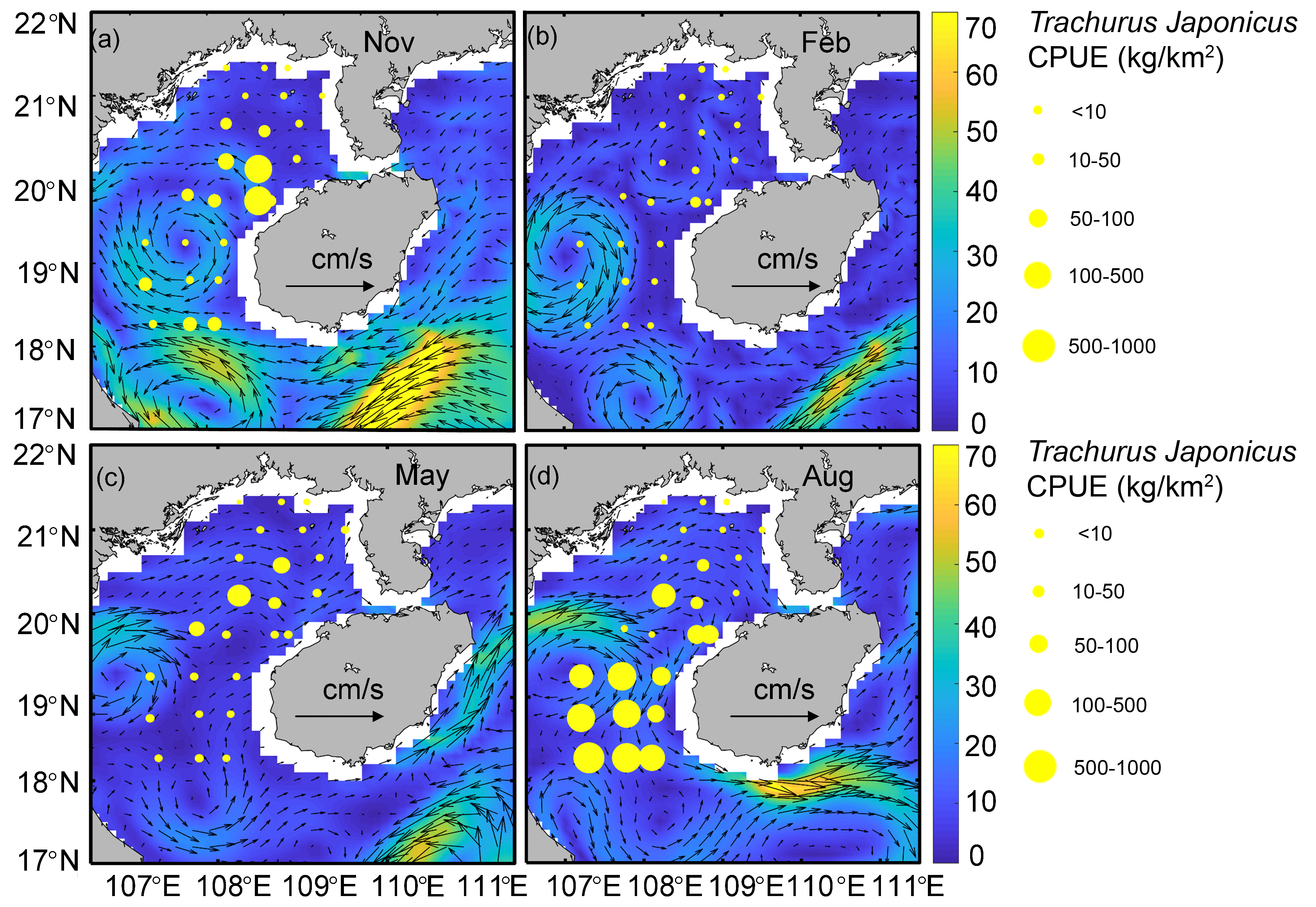

Supplement: Supplemental Information 3 — (A) Autumn. (B) Winter. (C) Spring. (D) Summer [file peerj-09-12337-s003.png]
